# A rare case of eight multiple primary malignant neoplasms in a female patient: A case report and review of the literature

**DOI:** 10.3892/ol.2014.2789

**Published:** 2014-12-10

**Authors:** JIEMIN ZHAO, YAN TAN, YUGANG WU, WEI ZHAO, JUN WU, MEI JI, LIANGRONG SHI, JINGTING JIANG, CHANGPING WU

**Affiliations:** 1 Department of Oncology, Third Affiliated Hospital of Suzhou University, Changzhou, Jiangsu 213003, P.R. China; 2 Department of Pathology, Third Affiliated Hospital of Suzhou University, Changzhou, Jiangsu 213003, P.R. China; 3 Department of Surgery, Third Affiliated Hospital of Suzhou University, Changzhou, Jiangsu 213003, P.R. China

**Keywords:** multiple primary malignant neoplasms

## Abstract

In recent years, the number of patients exhibiting multiple primary malignant neoplasms has been increasing. The current study reports the rare case of a 61-year-old Chinese female with eight histopathologically confirmed primary malignant neoplasms; to the best of our knowledge, such a high number of malignant neoplasms in one patient has not previously been described in the English literature. The tumors originated from the colon (five separate tumors over 32 years), the endometrium, the breast and the small intestine. It is important to diagnose multiple primary malignant neoplasms as such patients generally exhibit a more favorable prognosis compared with metastatic carcinoma patients. In addition, prolonged follow-up after surgery should be considered.

## Introduction

Multiple primary malignant neoplasms (MPMNs) may occur in a single organ or involve multiple organ systems and can be synchronous or metachronous. The current criteria for the diagnosis of MPMNs, which were established by Warren and Gates (1), are as follows: i) Each of the lesions must be malignant; ii) each of the lesions must exhibit distinctively different pathology; iii) and metastases from the prior malignancies must be excluded. Among patients with multiple primary malignancies, double cancers are commonly observed, triple cancers occur in 0.5% of these patients, and quadruple or quintuple cancers occur in <0.1% (2). The present study reports a rare case of a patient exhibiting eight MPMNs. Written informed consent was obtained from the patient.

## Case report

### Personal and family history

The present study describes the case of a 61-year-old female who attended the Third Affiliated Hospital of Suzhou University (Changzhou, China). The patient denied tobacco use or alcohol consumption when they first came to our hospital in 1979; however, the patient had been diagnosed with type two diabetes mellitus ~20 years ago and injects insulin to control blood glucose levels. One year prior to presentation, the patient’s older brother was diagnosed with brain glioma and two years prior; the patient’s nephew was diagnosed seminoma of the testis. In addition, the patient had a healthy 33-year-old daughter.

### Medical history

#### First tumor

**Figure 1:**
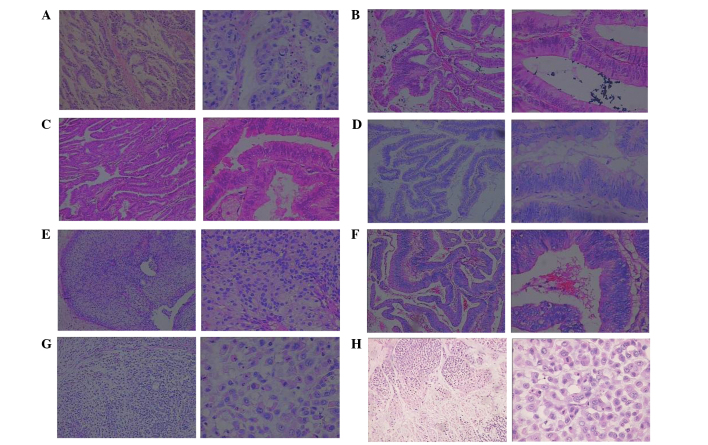
Photomicrographs of hematoxylin and eosin-stained specimens from the patient’s various carcinomas. (A) Grade III colonic adenocarcinoma (year, 1979); (B) grade II colonic adenocarcinoma (year, 1988); (C) well-differentiated endometrial adenocarcinoma (year, 1996); (D) colonic carcinoma (year, 1998); (E) canal papillary carcinoma of the right breast (year, 2004); (F) mushroom-like colonic adenocarcinoma (year, 2007); (G) colonic mucinous adenocarcinoma (year, 2011); (H) small intestinal mucinous adenocarcinoma (year, 2011). Within each figure part, the magnification of the left panel is ×100 and that of the right panel is ×400.

The first tumor identified was colon cancer, the initial symptoms of which were abdominal distension and diarrhoea. The patient visited our hospital and received a gastrointestinal barium meal and colonoscopy examination. The tumor was curatively resected in September 1979. Subsequent histopathological analysis identified a grade III (poorly differentiated) Dukes’ B2 adenocarcinoma of the ileocecal junction (Fig. 1A) (3), which, according to the tumor, node, metastasis (TNM) system was classified as T3N0M0, stage II (4). Following surgery, the patient underwent five cycles of chemotherapy with 5-fluorouracil [5-FU; 500 mg/m^2^ by intravenous (i.v.) infusion from day 1 to 5 every 21 days as a cycle], dactinomycin (8 μg/kg by i.v. infusion from day 1 to 5 every 21 days as a cycle) and thiotepa (0.2 mg/kg by i.v. infusion from day 1 to 5 every 21 days as a cycle).

#### Second tumor

In October 1988, an additional colon cancer was identified in the splenic flexure of the colon by routine endoscopic follow-up (Fig. 1B). The patient underwent curative surgery followed by five cycles of adjuvant chemotherapy with 5-FU (500 mg/m^2^ by i.v. infusion from day 1 to 5 every 21 days as a cycle), mitomycin (10 mg by i.v. infusion at day 1 every 21 days as a cycle) and vincristine (2 mg by i.v. infusion at day 1 every 21 days as a cycle).

#### Third tumor

In May 1996, the patient was diagnosed with cancer of the endometrium that had presented as vaginal hemorrhage. A hysterectomy was performed and pathological examination identified a well-differentiated, estrogen receptor (ER)- and progesterone receptor (PR)-positive adenocarcinoma of the endometrium (Fig. 1C).

#### Fourth tumor

In May 1998, an additional 2-cm-diameter tumor was identified in the descending colon by a routine colonoscopy, and the patient underwent descending colectomy. Subsequent pathological examination determined that the tumor was a descending colon polyp, which resembled an adenoma. Certain parts of the glandular epithelium demonstrated midrange atypical hyperplasia and malignant cells encroaching into the superficial muscular layer (Fig. 1D). The tumor was classified as T1N0M0, stage I.

#### Fifth tumor

In April 2004, a palpable mass was identified in the right breast and the patient was referred to the Department of Breast Surgery, the Third Affiliated Hospital of Suzhou University. Biopsy analysis indicated malignancy and the patient underwent a modified radical mastectomy with level II lymph node dissection. Pathological examination of the 1.0×1.0×0.8-cm resected mass identified canal papillary carcinoma with a clear resection margin (Fig. 1E). Additionally, the lymph nodes were negative for cancer; immunohistochemistry demonstrated that the patient was hadro-positive (+++) for ER, PR and p53, and negative for human epidermal growth factor receptor 2; and the tumor was classified as T1N0M0, stage I. This was the patient’s fifth malignancy. Following surgery, the patient received two cycles of chemotherapy with 5-FU (500 mg/m^2^ by i.v. infusion from day 1 to 5 every 21 days as a cycle), methotrexate (1 g/m^2^ by i.v. infusion at day 1 every 21 days as a cycle) and cyclophosphamide (600 mg/m^2^ by i.v. infusion at day 1 every 21 days as a cycle).

#### Sixth tumor

In May 2007 it was noted that the patient’s carcinoembryonic antigen (CEA) concentration was progressively increasing. The patient attended the hospital and received computed tomography (CT) and colonoscopy examinations. A 6-cm-diameter tumor was identified in the colon and palliative colectomy was performed. Subsequent pathological examination demonstrated a mushroom-like adenocarcinoma of the colon, the majority of which was mucinous adenocarcinoma (Fig. 1F) that encroached on the total depth of the intestinal wall. Immunohistochemistry was p53- and proliferating cell nuclear antigen-negative and nm23 -, cox-2- and epidermal growth factor receptor-positive. Following surgery, the patient underwent 12 cycles of chemotherapy with folinic acid (leucovorin), 5-FU and oxaliplatin. One cycle included i.v. leucovorin calcium at a dose of 200 mg/m^2^, bolus 5-FU at a dose of 400 mg/m^2^ and continuous i.v. 5-FU at a dose of 600 mg/m^2^ on day 1, followed by 85 mg/m^2^ oxaliplatin on day 2, and was repeated every 2 weeks.

#### Seventh and eighth tumors

In February 2011, the patient’s CEA concentration was again noted to be progressively increasing. Thus, two novel tumors were identified by positron emission tomography-CT and colonoscopy examination; one in the small intestine and one in the colon. The patient underwent partial small intestine and colon resection of a small intestinal tumor measuring 5.0×5.0×4.5 cm and a colonic tumor measuring 4.5×4.0×4.5 cm, respectively. Subsequent pathological examination demonstrated that the two tumors were poorly differentiated mucinous adenocarcinomas; however, the lymph nodes were negative for cancer (Fig. 1G). Following surgery, the patient received 12 cycles of chemotherapy with folinic acid, 5-FU and irinotecan. One cycle included i.v. leucovorin calcium at a dose of 200 mg/m^2^, bolus 5-FU at a dose of 400 mg/m^2^ and continuous i.v. 5-FU at a dose of 600 mg/m^2^ on day 1, followed by 180 mg/m^2^ irinotecan on day 2, and was repeated every 2 weeks. The last chemotherapy cycle ended in September 2011, and the patient remained alive and appeared to be tumor-free at the time of writing.

## Discussion

The current study presents the case of a patient with eight pathologically verified primary tumors originating in the colon, endometrium, breast and small intestine, including six of the digestive system. According to Kapsinow’s definitions (5), the first six tumors were metachronous carcinomas and the most recent two tumors were synchronous carcinomas. Cases of multiple primary malignancies have been increasingly reported in recent years (6–9), however, it remains rare to encounter a patient with eight primary malignancies. As six of the tumors were located in the intestinal tract, endoscopies were performed on direct relatives of the patient, including the patient’s daughter, nephew and two brothers; however, no familial adenomatous polyposis or other hereditary nonpolyposis colorectal cancer were identified.

Pathologists were recruited to aid in determining whether the colonic tumors of the present patient were primary or metastatic. The following are crucial characteristics for differentiating between the two types of tumor: i) The majority of MPMNs are located in the wall of the colon and rectum, whereas metastatic carcinomas generally occur adjacent to the colon and rectum or in the regional lymph nodes; ii) the majority of newly diagnosed MPMNs are located in a different region to previous tumors, whereas relapsing carcinomas commonly occur at the site of the primary tumor; iii) the majority of newly diagnosed MPMNs are singular, whereas metastatic carcinomas tend to be multiple; iv) synchronous multiple primary carcinomas of the colon and rectum may exhibit similar or different pathology to the primary tumor, whereas metastatic carcinomas exhibit similar pathology to the primary tumor (9); and v) the diagnosis of synchronous MPMNs of the colon and rectum requires that tumors be separated by >5 cm of normal colonic and rectal wall (10). In addition, according to a previous report (11), synchronous and metachronous primary advanced colorectal carcinomas in the same patient may exhibit variable KRAS, NRAS and BRAF genotypes, which have diagnostic and therapeutic implications when metastases occur, and may facilitate the identification of simultaneous or metachronous distant metastases.

Although the mechanisms responsible for the development of MPMNs are yet to be fully elucidated, frequently implicated factors include genetic susceptibility, immune status and previous intensive exposure to carcinogens, such as chemo- and/or radio-therapy used to treat tumors (7,12). Furthermore, a number of hereditary conditions are associated with multiple primary malignant neoplasms; for example, Li-Fraumeni syndrome is a rare disorder that greatly increases the risk of developing various types of cancer. The types of cancer most often associated with this syndrome are breast cancer, osteosarcoma and soft tissue sarcomas, as well as brain tumors, leukemias and adrenocortical carcinoma (13). In addition, the CHEK2 and TP53 genes are associated with Li-Fraumeni syndrome, as >50% of families with this syndrome exhibit inherited TP53 gene mutations (14). The present study used immunohistochemistry to identify mutations in the TP53 gene in each of the current patient’s tumors, however, the results were consistently negative. Considering the large numbers of primary tumors the patient developed, it was proposed that an unidentified mutation may exist and, thus, the patient was at a high risk of developing additional tumors in the future.

Novel technologies are able to identify various genetic changes, such as punctiform mutations, loss of heterozygosity or genetic instability. Furthermore, microsatellite instability reportedly occurs more frequently and has a worse prognosis in cases of MPMNs compared with in sporadic cancers (15). Similar epigenomic and epigenetic events are frequently observed within a pair of synchronous cancers, indicating the presence of a field defect; for example, microsatellite instability in sporadic cancer is typically due to epigenetic silencing of the MLH1 gene (16).

Various studies have demonstrated that chemo- and radio-therapy are carcinogenic (17,18). By affecting the function and synthesis of DNA, a variety of chemotherapeutic agents, particularly alkylating agents, may cause damage to dividing cells in healthy tissues, such as bone marrow or gastrointestinal mucosa cells. The patient described in the present report received chemotherapy numerous times and this may have contributed to the development of additional MPMNs.

The literature regarding MPMNs recommends that secondary primary cancers should be resected as early as possible and concludes that the probability of successful treatment is similar to that of a single cancer (19). Furthermore, long-term follow-up and screening strategies appear to be important for patients who have undergone curative resection of malignancies. Healthcare workers should consider that the prevalence of multiple primary malignancies appears to be increasing and, thus, should be prepared to differentiate secondary primary cancer from metastatic cancer. The present patient was cooperative and attended the hospital frequently; therefore, all of the tumors were diagnosed early and removed.

Koutsopoulos *et al* (20) reviewed cases with three or more primary malignancies. The occurrence of three primary malignancies in one patient is not common, however, neither is it particularly rare. Therefore, it is important that patients who have been treated for cancer are provided with adequate follow-up care. Upon the appearance of the symptoms and signs of a tumor in a patient who has previously been treated for a primary cancer, metastasis should not be assumed as the diagnosis. Instead, the possibility of a localized and curable secondary primary cancer should be considered and evaluated, and tumor markers or positron emission tomography and computed tomography may be useful for follow-up. Although the present patient developed a large number of tumors over a >32-year period, the patient is alive and has a good quality of life. Therefore, multiplicity of primary malignancies itself does not necessarily indicate a poor prognosis provided that adequate diagnosis and treatment are provided.

In conclusion, it is important for health-care workers to consider that the appearance of an additional tumor in a cancer patient may be either a metastatic or novel lesion, and the possibility of a metachronous or a synchronous malignancy should be investigated. Furthermore, prolonged follow-up after surgery should be considered.
